# Supplementary figures and images for: CRISPRi screen of long non-coding RNAs identifies LINC03045 regulating glioblastoma invasion
Source: PLoS Genet. 2024 Jun 10;20(6):e1011314. doi: 10.1371/journal.pgen.1011314 (PMC11192328; doi:10.1371/journal.pgen.1011314)

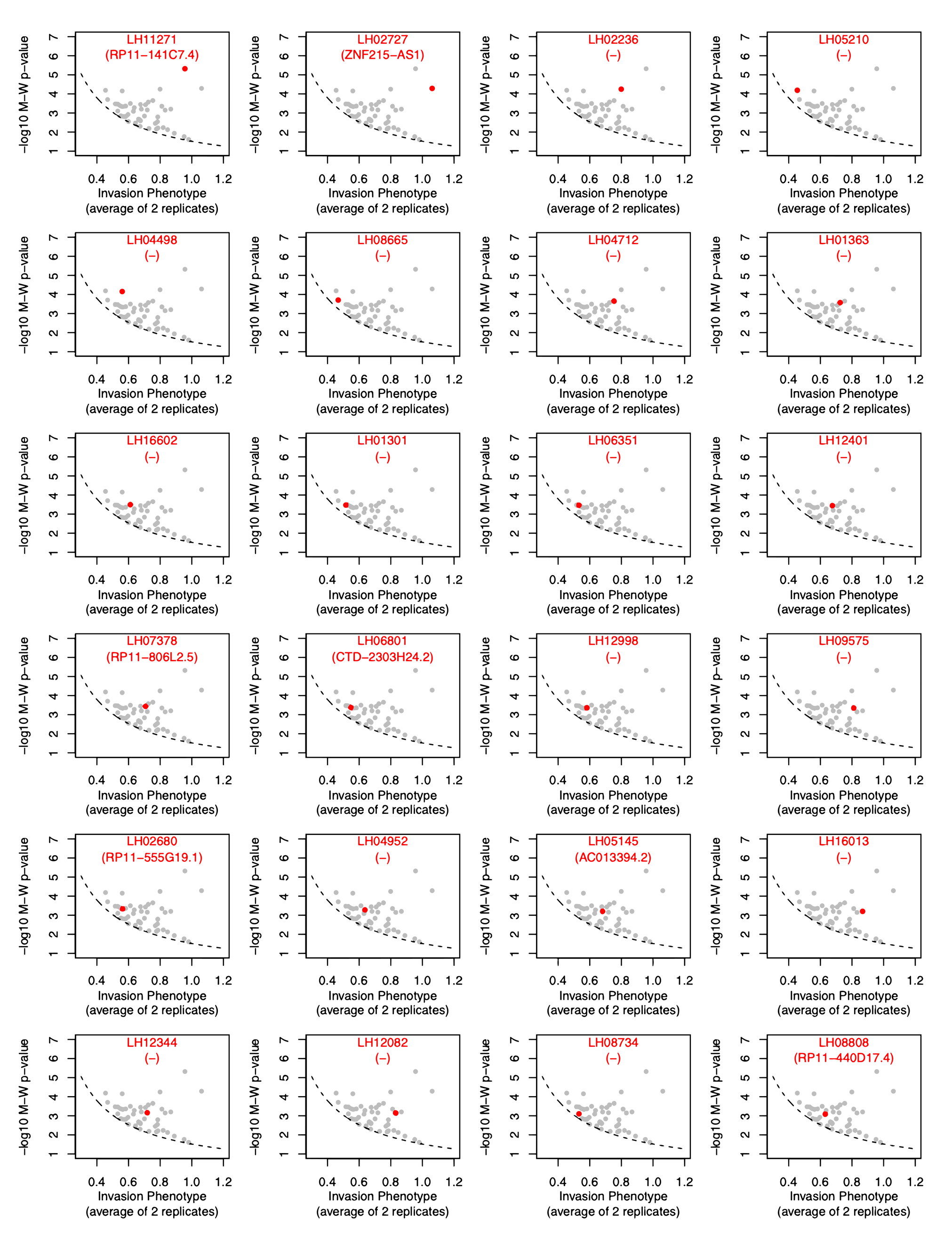

Supplement: S1 Fig — Screen replicates were averaged and the top 3 sgRNAs for each lncRNA compared to non-targeting controls were used to determine screen hits. The dashed lines represent thresholds to determine screen hits. (TIF) [file pgen.1011314.s001.tif]

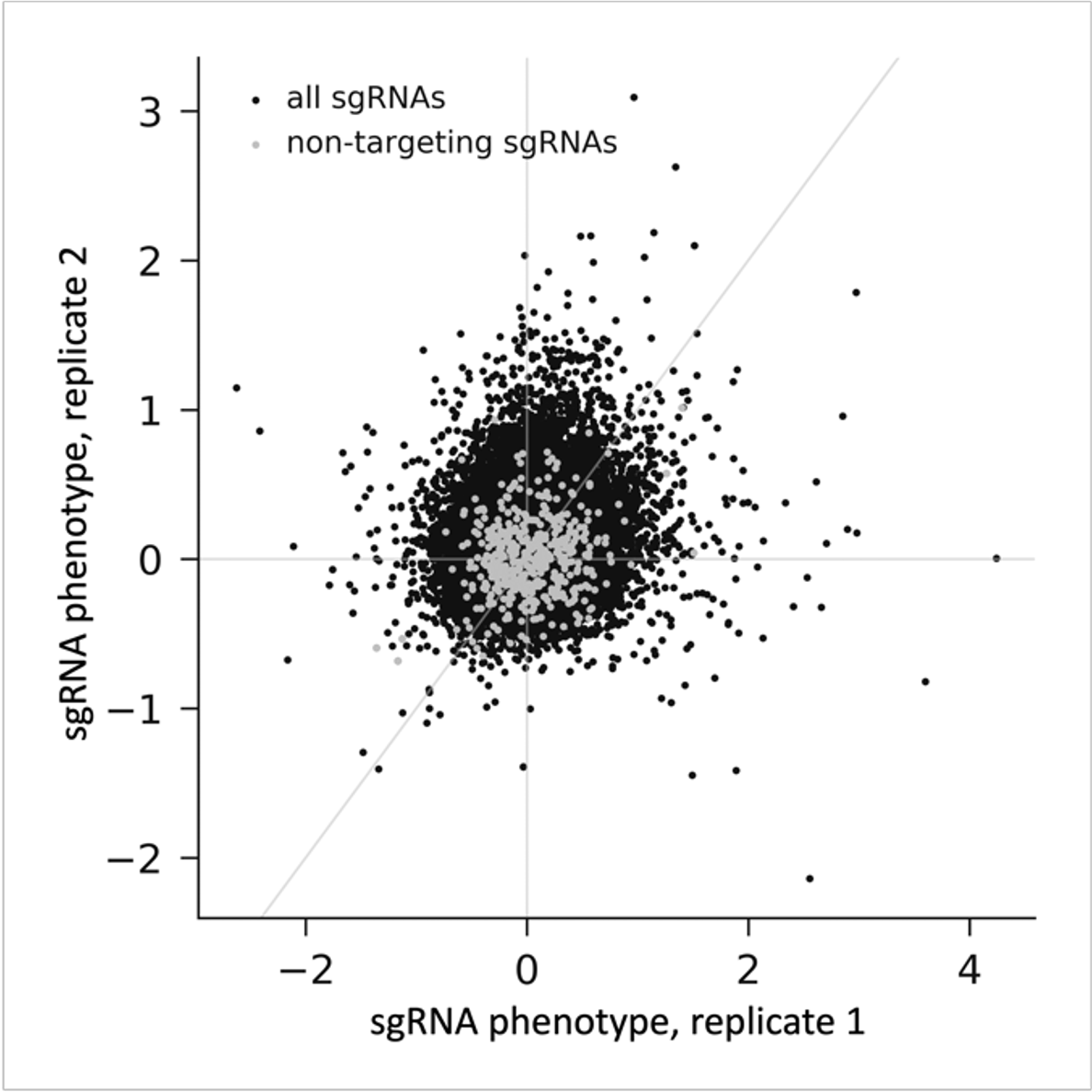

Supplement: S2 Fig — A positive phenotype indicates knockdown of a lncRNA caused a decrease in invasion, whil a negative phenotype indicates knockdown of a lncRNA caused an increase in invasion. The X and Y-axes represent different screen replicates. (TIFF) [file pgen.1011314.s002.tiff]

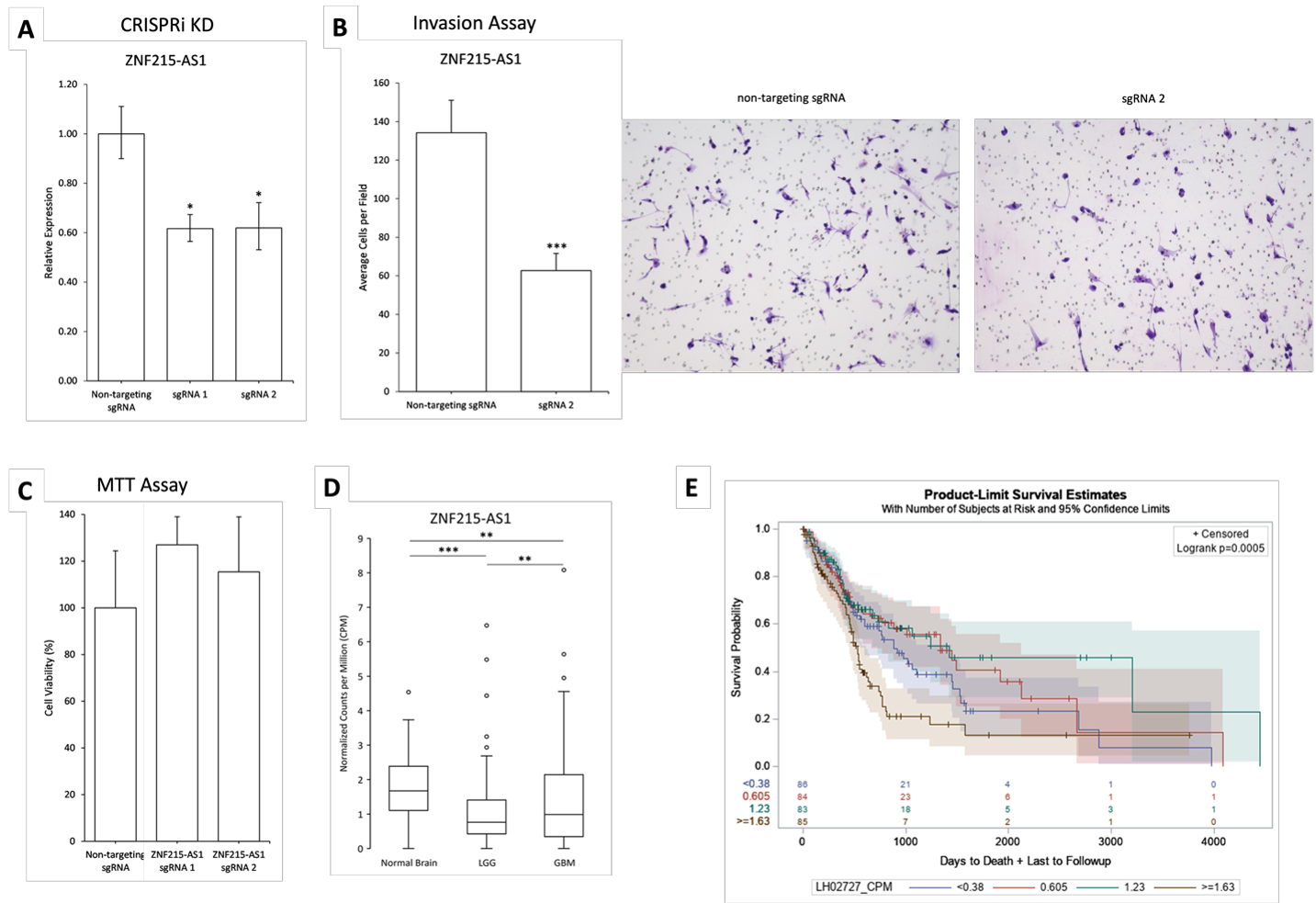

Supplement: S3 Fig — A) qPCR of CRISPRi KD of the top two sgRNAs for ZNF215-AS1 in U87 cells. All expression levels normalized to the housekeeping gene RPLP0. KD cells compared to the non-targeting sgRNA. Data expressed as mean ± SD for replicates. B) Representative images of invasion assay through a Matrigel-coated Boyden chamber after KD of ZNF215-AS1 by CRISPRi KD in U87 cells. The bar graph represents the number of invaded cells per field counted, with KD cells compared to the non-targeting sgRNA. Data are expressed as mean ± SEM of three independent experiments with 9 fields imaged per experiment. C) An MTT assay showing no change in proliferation after KD of ZNF215-AS1 or LH02236 by CRISPRi in U87 cells. KD cells compared to the non-targeting sgRNA. Data are expressed as mean ± SD. *p ≤ 0.05, **p ≤ 0.01, ***p ≤ 0.001 D) Boxplots of pairwise comparisons of ZNF215-AS1 expression in normal human brain cortex to low grade glioma (LGG) and glioblastoma (GBM) patient samples. E) Kaplan-Meier survival curves of ZNF215-AS1 for gliomas (stages 1–4) with expression quantified by normalized counts per million (CPM). Position coordinates were used to identify both genes in RNA-seq data. Expression was normalized to standardized genes on the same chromosome. Censored data indicates patients lost to follow-up. *p ≤ 0.05, **p ≤ 0.01, ***p ≤ 0.001 (TIFF) [file pgen.1011314.s003.tiff]

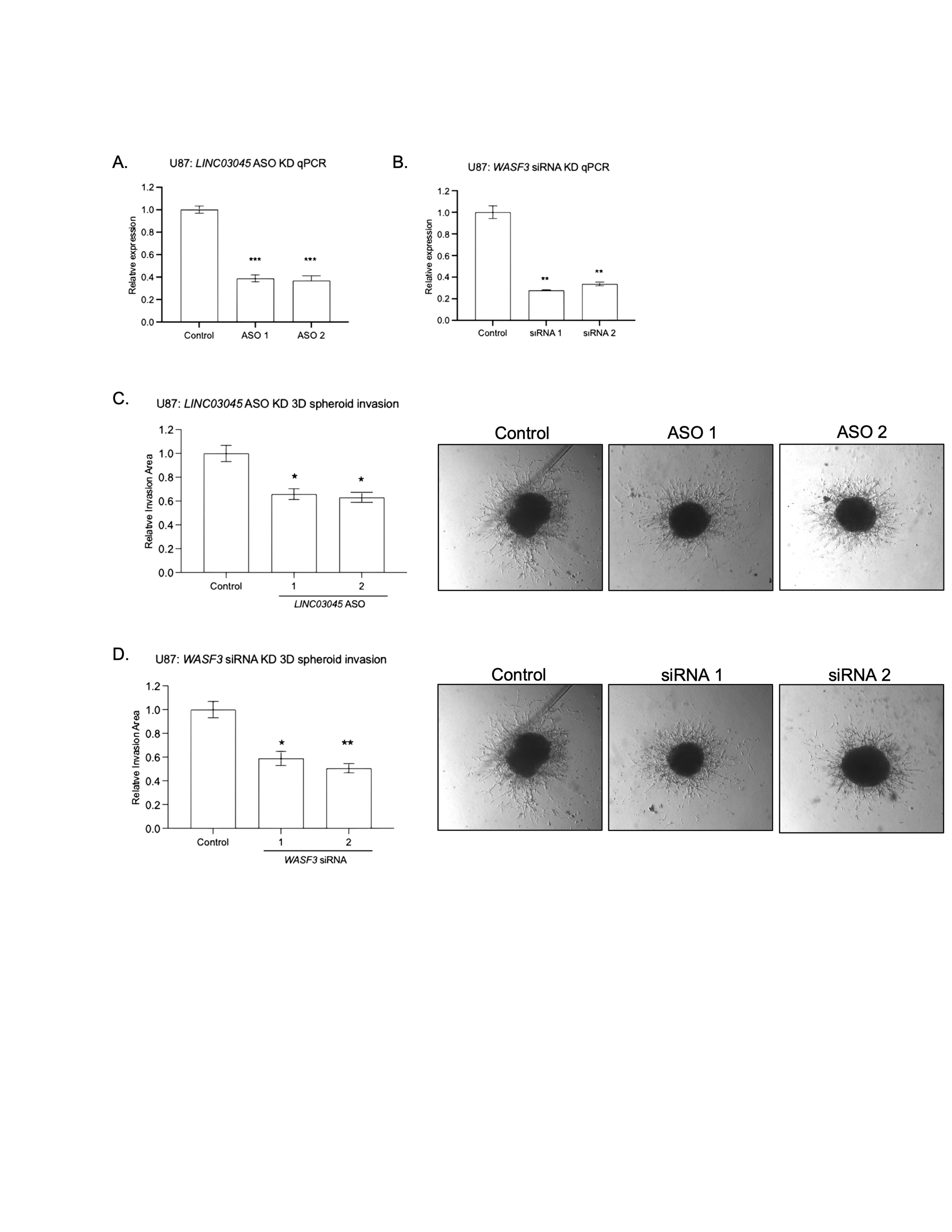

Supplement: S4 Fig — qPCR confirmation of LINC03045 KD (A) and WASF3 KD (B) in U87 cells relative to control untransfected U87 cells, after transfection with LINC03045 ASO or WASF3 siRNA respectively. All data were normalized to RPLPO housekeeping gene. Data are expressed as mean ± SEM for replicates. *p ≤ 0.05, **p ≤ 0.01, ***p ≤ 0.001. (C) Quantification and representative images of U87 spheroid invasion into surrounding invasion matrix after ASO-mediated knockdown of LINC03045 compared to control untransfected U87 spheroids. Data are expressed as mean ± SEM for replicates. *p ≤ 0.05, **p ≤ 0.01, ***p ≤ 0.001. (D) Quantification and representative images of U87 spheroid invasion into surrounding invasion matrix after siRNA-mediated knockdown of WASF3 compared to control untransfected U87 spheroids. Data are expressed as mean ± SEM for replicates. *p ≤ 0.05, **p ≤ 0.01, ***p ≤ 0.001. Spheroid images taken at 2x magnification. (TIF) [file pgen.1011314.s004.tif]

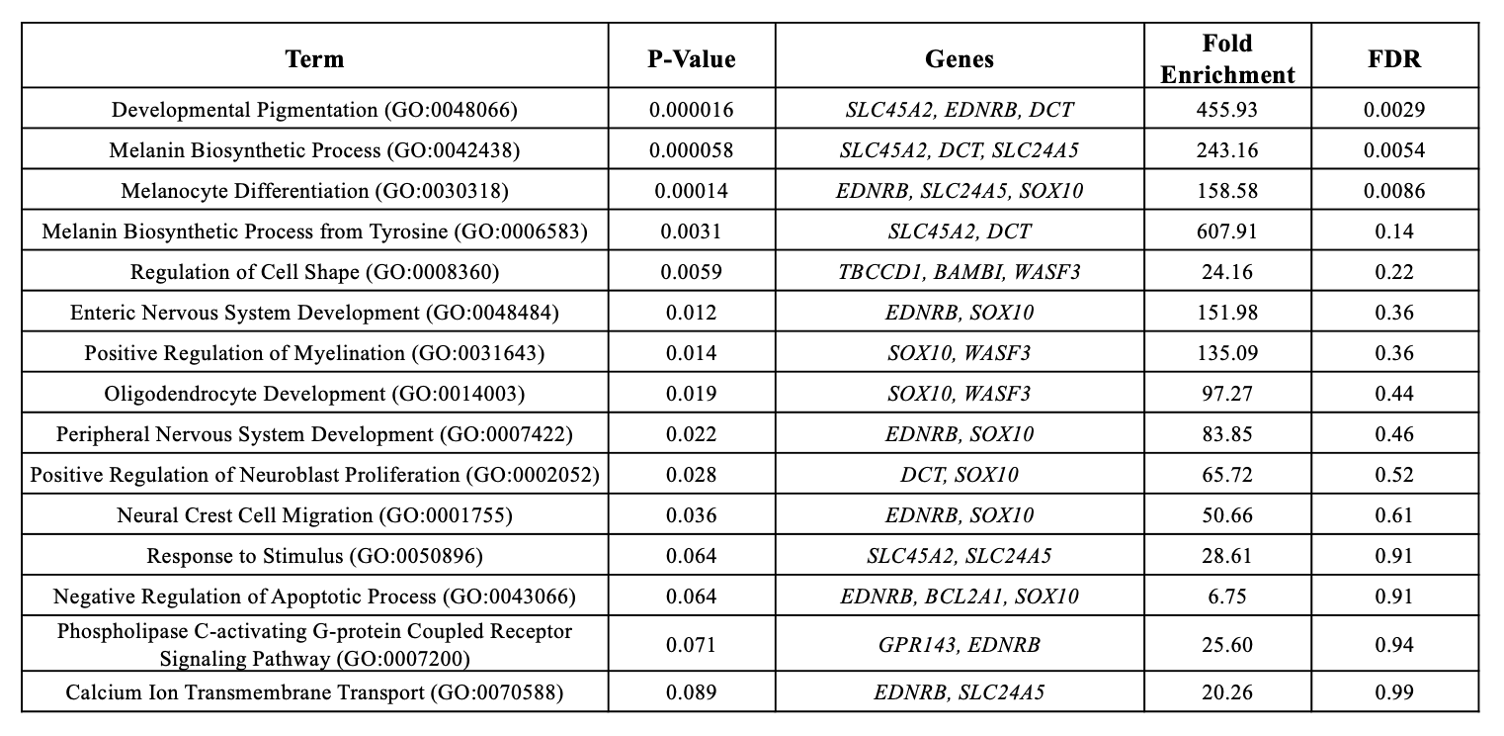

Supplement: S1 Table — (TIFF) [file pgen.1011314.s005.tiff]
