# Supplementary material for: CRISPRi screen of long non-coding RNAs identifies LINC03045 regulating glioblastoma invasion
Source: PLoS Genet. 2024 Jun 10;20(6):e1011314. doi: 10.1371/journal.pgen.1011314 (PMC11192328; doi:10.1371/journal.pgen.1011314)
Supplement: Attachment — Submitted filename: Comments.docx [file pgen.1011314.s006.docx]

In this study, the authors employed the CRISPRi screen method to identify a novel candidate associated with the invasion property in a glioma cell line. They have effectively demonstrated the reproducibility of their invasive assay and have proposed a potential drug candidate for future glioma treatment. The authors conducted CRISPRi screening in the U87 cell line, selected a candidate, and subsequently validated it in three different cell lines, including a patient-derived glioma cell line. However, in order to bolster the reliability of the results, it is imperative to conduct screening experiments in additional cell lines, ensuring that they exhibit validated invasive properties.

Major Comments:

1.	To begin with, it is essential to assess the invasiveness of the U87 commercial cell line, potentially through a 3D invasion assay. Given that this cell line may not precisely reflect glioblastoma behavior in real-world scenarios, many researchers prefer employing patient-derived cell lines or tumor spheres. Considering the relatively short duration required for CRISPRi screening, it is advisable to conduct the screening in another cell line, such as USC02 (utilized in the validation experiment), while also demonstrating the invasiveness of this cell line.

2.	In Figure 1, the y-axis appears to represent -log P-values, while the x-axis likely represents log fold changes when compared to nontargeting guide RNAs. ZNF215-AS1 emerges as a seemingly more significant candidate. Could the authors provide a rationale for choosing the LINC03045 candidate over ZNF215-AS1? Additionally, in the context of "Positive hits were identified through this process," please specify the positive hits in both the figure and the manuscript. Furthermore, when mentioning, "The dashed lines represent thresholds to determine screen hits," it would be beneficial to elucidate the principle governing the selection of these thresholds. Typically, in cases where the y-axis represents -log P-values, a dashed line at -log(0.05) is used to signify a threshold, with a p-value less than 0.05 indicating a significant finding, as stated in the method section.

Minor Comments:

1.	In Figure 1, it is advisable to include a correlation plot illustrating the guide enrichment between the two replicates. This addition would enhance the reliability of the results.

2.	Consider converting most of the images into vector format, particularly for figures depicting heatmaps or volcanoplots. This change would improve the clarity and resolution of these figures.
