# Supplementary material for: CRISPRi screen of long non-coding RNAs identifies LINC03045 regulating glioblastoma invasion
Source: PLoS Genet. 2024 Jun 10;20(6):e1011314. doi: 10.1371/journal.pgen.1011314 (PMC11192328; doi:10.1371/journal.pgen.1011314)
Supplement: Attachment — Submitted filename: Reviewer Responses Final.docx [file pgen.1011314.s007.docx]

**Title of the Manuscript**: CRISPRi Screen of Long Non-coding RNAs Identifies *LINC03045* Regulating Glioblastoma Invasion

**Manuscript Number**: PGENETICS-D-23-00531

***Reviewer 1*:** The authors seek to identify the role of lncRNAs in GBM cell invasion. The long non-coding RNA LINC03045 is identified as a positive hit in a CRISPRi screen using the U87 GBM cell line in an in vitro invasion assay. This result is validated utilizing ASO/CRISPRi KD in vitro with commercially available and patient-derived GBM cell lines. Clinically relevant correlation is shown with TCGA and GTEx analysis demonstrating a relationship between LINC03045 expression and patient outcomes. A further positive association is demonstrated between LINC03045 and WASF3 expression in vitro and in patient cohorts, as well as epistasis through in vitro invasion.

**Major Points**

**Comment 1**: The study results and conclusion would be most prominently strengthened with some in vivo validation demonstrating either a change in invasion phenotype or animal survival.

**Authors’ Response**:

- This is an excellent point, as additional phenotype data would strengthen our results. We first conducted multiple attempts at *in vivo* knockdowns over a prolonged period. However, we encountered challenges maintaining stable lentiviral *LINC03045* lentivirus knockdown in our patient-derived cell model, with cells losing plasmid fluorescence over the required time for the assay.
- To provide an assay with physiologic relevance that was closer to an *in vivo* model, we conducted 3D invasion assays, where we would be able to provide continuous knockdown of *LINC03045* via ASO, without the challenge of stable lentiviral transduction required *in vivo*. The choice of using these 3D models was in line with recent studies that have also chosen the same model for assessment of phenotype approximating *in vivo* behavior (Hira, *Biotech*). Our 3D assays do successfully corroborate our prior *in vitro* data, demonstrating decreased 3-dimensional invasion of glioma cells with both *LINC03045* and *WASF3* knockdown compared to untreated controls.
- We have added these new results to our METHODS, RESULTS, and SUPPLEMENTAL FIGURE 4. Further, the need for a study of true *in vivo* knockdown is highlighted in the limitations section of our discussion.

**Comment 2**: Figure 2. Survival curves should be controlled for grade and IDH status. It is not really relevant to compare lower grade gliomas (which are likely to be IDH mutant) to IDH WT GBMs. In other words, all tumors of a certain grade (you can stick to GBM) should be stratified by LINC03045 expression to determine if expression of this lncRNA is associated with worse prognosis within a specific grade and molecular subtype.

**Authors’ Response**:

- We agree with this point and we now have included multiple additional analyses adjusting both for glioma grade and IDH status in Cox regression analysis. In line with the specific example provided by the reviewer, we have now included additional survival analyses of grade 4 gliomas adjusting for IDH status. *LINC03045* expression was not significantly associated with survival among Grade 4 gliomas (p=0.40, Fig 2c).
- Because the TCGA stratifies lower grade gliomas (grade 2 and 3) together due to the nonspecific grading data on 20 tumors (grade 2 or grade 3 only), we also included an IDH-adjusted survival analysis of this cohort. Analysis of this lower grade glioma cohort (when including the 20 uncertain grade 2/3 patients) did show that *LINC03045*was associated with survival (p=0.02, Fig 2c). Notably, grade 2 and 3 gliomas within this cohort did not significantly differ in their *LINC03045*expression.
- However, we also conducted analysis of Grade 2 gliomas alone when excluding the 20 patients with uncertain grade 2/3 pathology. *LINC03045*expression was not associated with survival in this cohort (p=0.26, data not shown).
- Despite the potential association between *LINC03045* expression and survival in the lower grade glioma cohort, it does appear that when strictly stratifying within grade 4 tumors and adjusting for IDH status, that *LINC03045* is not associated with survival. While this may be due to the extremely low baseline expression of *LINC03045* and the available powering, we have now clarified in RESULTS, DISCUSSION and FIGURE 2.

**Comment 3**: Figure 3. I am somewhat surprised by the dramatic in vitro invasion phenotype despite a relatively modest inhibition of RNA expression. Do the authors have any thoughts as to why?

**Authors’ Response**:

- This is also an excellent point. LncRNA knockdown is challenging by both ASO and CRISPRi, with our knockdowns in the experiments ranging from 40-70%. However, it is accurate that we did notice dramatic invasion changes of up to 80% in the Matrigel invasion based on knockdown of less than 40%. Essential genes in certain processes have been previously noted to show exaggerated phenotype with small changes in gene expression.(Mukherjee, *mSystems*, 2021; Garfield, *Bioscience*, 2010)
- Notably, lncRNAs are commonly defined by an abundance of expression in a small subset of cells, often being referred to as extremely abundant and specific. (Liu, *Genome* *Biology*, 2016). It may be that gene expression only at high levels in an aggressive, invading, lncRNA-rich subpopulation is capable of demonstrating invasion at 24 hours, and thus our more modest 40% knockdown affects the 24 hour invasion phenotype. We have now included a discussion on this surprising finding in our DISCUSSION Section.

Comment 4: A more thorough comparison should be made of the in vivo LINC03045-associated gene expression changes and the in vitro LINC03045 KD-associated changes. Also is here a relationship between WASF3 and any of the GO or KEGG terms that are enriched in the in vivo gene expression analyses?

**Authors’ Response**:

- This is an excellent point and we have done additional gene expression analyses to evaluate the points noted. We first conducted additional comparison of *in vivo* *LINC03045* associated gene expression changes and *in vitro* *LINC03045* KD associated changes by evaluating the intersection of differentially expressed genes. We now include a Venn diagram demonstrating that 16 of the 36 significantly differentially expressed genes in the lncRNA KD overlap with genes associated with the TCGA. For the TCGA-associated genes, we specifically included protein coding genes that show a significant association with *LINC03045*. Overlapping genes are involved in multiple notable pathways including regulation of cell shape and neural crest migration. This is now included in our METHODS, RESULTS, DISCUSSION and FIGURE 5G, with involved genes noted in Supplemental Table 1.
- We also followed the reviewer’s suggestion to evaluate the relationship between WASF3 and GO terms enriched in the TCGA in vivo analysis. We specifically evaluated enriched GO terms of *LINC03045*correlated genes for the presence of WASF3. Notably, the GO term for cytoskeleton organization (GO:0007010) process was enriched. It does appear that cytoskeleton organization/cell shape is heavily involved in both the *in vitro* KD results and the *in vivo* TCGA *LINC03045* associated genes. This follows the literature noting cytoskeletal reorganization is commonly associated with invading tumor cells. We have now also included this data and prior literature within our METHODS, RESULTS and DISCUSSION sections.

**Comment 5**: Do the authors know how mechanistically LINC03045 triggers WASF3 expression?

- This is a critical point. While formal mechanistic studies involving co-localization or RNA pulldown were not performed, there are hypotheses as to the potential mechanism. LncRNAs commonly exert function via interaction with transcriptional regulators, functioning as guides or scaffolds for transcription factors. Unfortunately, the transcriptional regulation of WASF3 remains relatively unstudied, and this would require extensive further study.

- Another possibility that would have to be pursued in subsequent experiments is the possibility of lncRNAs acting as microRNA sponges. Using the microRNA targetSCAN tool, multiple miRNAs are noted to bind WASF3, including miR-130a and miR-23a, miR-23b, and miR 23c. MicroRNAs often require as few as 6 nucleotides to bind, and the 7-nt WASF3 binding site for all 4 microRNAs also binds 6 of 7 consecutive nucleotides on *LINC03045*.
- Ultimately, however we do not have a specific mechanistic data, and thus this is a limitation and requirement for further study. We have included this in the limitations section of our DISCUSSION.

**Comment 6**: Figure 7. Two different siRNAs should be used for WASF3 KD experiments.

**Authors’ Response**:

- We agree and we have now included phenotype experiments for WASF3 KD using two separate siRNAs, including results in Supplemental Figure 4, with both knockdowns showing significant qPCR transcriptional decrease and 40-50% knockdown of invasion phenotype. This has now been included in the METHODS, RESULTS, and SUPPLEMENTAL FIGURE 4.

**Comment 7**: In epistasis experiments (Fig 8), WASF3 overexpression alone should be presented in the invasion assay.

**Authors’ Response**: We have included results with WASF3 expression alone in the invasion assay.

- We agree this is necessary and now have included this in Figure 8. Results are now included in the METHODS, RESULTS and FIGURE 8.

**Minor Points**

**Comment 1**: As the steps in Fig 8A are not proven in the manuscript, it should be shown in the figure and discussed in the manuscript in a more tempered manner. There is no evidence that STAT3 is involved here and that LINC03045 directly regulates WASF3 expression.

**Authors’ Response**: This is a well-made point, and it is very true that our mechanism has yet to be defined clearly enough regarding the involvement of STAT, or whether *LINC03045* directly regulates WASF3 transcription. Further, whether the effects of *LINC03045* upon WASF3 directly regulates the JAK/STAT/WASF3 complex is also unknown. After extensive discussion among our group, we have decided to remove the figure to avoid any assumptions by readers. We have also significantly tempered the discussion regarding the relationship of *LINC03045* with WASF3 and STAT throughout the manuscript and particularly in the ABSTRACT, INTRODUCTION, and DISCUSSION sections.

***Reviewer 2*:** In this study, the authors employed the CRISPRi screen method to identify a novel candidate associated with the invasion property in a glioma cell line. They have effectively demonstrated the reproducibility of their invasive assay and have proposed a potential drug candidate for future glioma treatment. The authors conducted CRISPRi screening in the U87 cell line, selected a candidate, and subsequently validated it in three different cell lines, including a patient-derived glioma cell line. However, in order to bolster the reliability of the results, it is imperative to conduct screening experiments in additional cell lines, ensuring that they exhibit validated invasive properties.

**Major Points**

**Comment 1**: To begin with, it is essential to assess the invasiveness of the U87 commercial cell line, potentially through a 3D invasion assay. Given that this cell line may not precisely reflect glioblastoma behavior in real-world scenarios, many researchers prefer employing patient-derived cell lines or tumor spheres. Considering the relatively short duration required for CRISPRi screening, it is advisable to conduct the screening in another cell line, such as USC02 (utilized in the validation experiment), while also demonstrating the invasiveness of this cell line.

**Author’s Response**: This is an excellent point and we have attempted to replicate our screen in patient derived samples, particularly in the USC02 patient derived line used in our manuscript. Unfortunately, while the reviewer makes an excellent point regarding the shortened 24-hour screen, cells in our screening method still require a week of transfection and selection prior to the screen. We found that stable transfection of both the dCas9 and sgRNA plasmids with the number of sgRNA variations required for the screen was much more difficult in the patient derived cell line (versus U87), with loss of transfection presenting a significant challenge. While prior screening manuscripts have successfully employed patient derived cell lines in screens as perceptively suggested by the reviewer, we do note that our encountered limitation has also limited several prior glioma screens to U87 as well. As noted above, we did include a 3D invasion assay as well as validation in the patient derived line to attempt to improve the applicability of our results. However, we agree that this is a significant limitation to our work and have extensively highlighted this in the limitations section of our DISCUSSION.

**Comment 2**: In Figure 1, the y-axis appears to represent -log P-values, while the x-axis likely represents log fold changes when compared to nontargeting guide RNAs. ZNF215-AS1 emerges as a seemingly more significant candidate. Could the authors provide a rationale for choosing the LINC03045 candidate over ZNF215-AS1? Additionally, in the context of "Positive hits were identified through this process," please specify the positive hits in both the figure and the manuscript. Furthermore, when mentioning, "The dashed lines represent thresholds to determine screen hits," it would be beneficial to elucidate the principle governing the selection of these thresholds. Typically, in cases where the y-axis represents -log P-values, a dashed line at -log(0.05) is used to signify a threshold, with a p-value less than 0.05 indicating a significant finding, as stated in the method section.

**Author’s Response**:

- This is an excellent point. We initially chose *LINC03045* over ZNF215-AS1 due to a less clear association between gene expression and tumor grade/patient survival. However, it is true that ZNF215-AS1 does show a strong phenotype in the screen. We have now included additional experiments more completely evaluating both the clinical phenotype of ZNF215-AS1 in patient data, as well as additionally including knockdown of ZNF215 and resulting phenotypes. This data does show that ZNF215-AS1 may actually represent a secondary promising lncRNA for study as well despite the more modest clinical response, though the highest quartile of gene expression does associate with patient survival. We have now included an extensive additional figure including lncRNA KD and invasion phenotype, MTT assay after KD, and association with tumor grade and patient survival.
- We agree it is important to highlight all positive hits and we have now included a supplemental highlighting each specific hit and the associated z-score. While multiple hits were unannotated due to limited data on lncRNAs, the previously annotated genes are also included. This is now included in the RESULTS and SUPPLEMENTAL FIGURE 1.
- We agree that it is important to clarify the principles governing selection of thresholds for identification of positive hits. We followed methods of prior screens, and exactly as noted by the reviewer, the threshold description of -log(0.05) signified a threshold and was used for the y-axis cutoff. The effect size portion of the score (with a minimum effect size of greater than ~25% at the lowest p-values) was also adopted from previous methods, with the screen score incorporating both p-value and effect size was also chosen based on prior study methodology, x-log_10_ p value (Liu, Science, 2017).

**Minor Points**

**Comment 1**: In Figure 1, it is advisable to include a correlation plot illustrating the guide enrichment between the two replicates. This addition would enhance the reliability of the results.

**Author’s Response**: We agree and we have now included a correlation plot illustrating the guide enrichment between the two replicates to enhance the reliability of the results. This is now included in the METHODS, RESULTS, and SUPPLEMENTAL FIGURE 2.

**Comment 2**: Consider converting most of the images into vector format, particularly for figures depicting heatmaps or volcano plots. This change would improve the clarity and resolution of these figures.

**Author’s Response**: This is an excellent point and we have converted the images into vector format, ensuring that all heatmaps and volcano plots are converted, to improve the clarity and resolution of these figures.
