# Supplementary material for: CRISPRi screen of long non-coding RNAs identifies LINC03045 regulating glioblastoma invasion
Source: PLoS Genet. 2024 Jun 10;20(6):e1011314. doi: 10.1371/journal.pgen.1011314 (PMC11192328; doi:10.1371/journal.pgen.1011314)
Supplement: Attachment — Submitted filename: Review_ksg.docx [file pgen.1011314.s008.docx]

Compared to the initial manuscript, the authors have significantly enhanced the quality and presentation of their results. I have a few comments and suggestions for further improvement.

Firstly, I recommend that the authors provide a clean version of the manuscript, as the track-changed format can be difficult for reviewers to read and evaluate.

Secondly, the authors mentioned the challenges of using Cas9-based techniques in patient-derived cell lines. While this can indeed be challenging, I would like to suggest considering lentivirus-based screening methods. In my experience, this approach has been effective with glioma patient-derived cell lines and could potentially be beneficial for their CRISPR-based screening experiments.

Thirdly, the correlation scatter plot in the supplementary figure appears to show a low extent of correlation. It would be helpful if the authors included the correlation coefficient, such as Spearman's or Pearson's, to quantify the correlation and provide a clearer interpretation of the data.

Overall, I believe the manuscript is now suitable for publication.
